# Potential overtreatment in elderly patients with diabetes mellitus: Results from a cross-sectional study in German general practice

**DOI:** 10.1080/13814788.2024.2447723

**Published:** 2025-02-18

**Authors:** Solveig Weise, Christiane Oelschläger, Susanne Unverzagt, Jens Abendroth, Marcus Heise, Thomas Frese

**Affiliations:** Institute of General Practice and Family Medicine, Medical Faculty, Martin-Luther-University Halle-Wittenberg, Halle, Saale, Germany

**Keywords:** Elderly, diabetes mellitus type 2, general practice, overtreatment, cross-sectional studies

## Abstract

**Background:**

It is important for general practitioners (GPs) to protect elderly patients with diagnosis of diabetes type 2 (DM2) from overtreatment.

**Objective:**

To analyse the metabolic control and treatment of elderly patients with DM2 in general practices.

**Methods:**

This cross-sectional study involved 46 general practices in a federal state of Germany. Inclusion criteria for patients were diagnosis of DM2, age of 70 years or above, no palliative care and at least one practice contact within the last six months. A study nurse randomly selected 10 eligible patients and extracted data on haemoglobin A1c (HbA1c), diabetes treatment, secondary prevention and GP’s characteristics. Risk of overtreatment was defined as having a HbA1c <47.5 mmol/mol (6.5%) and receiving glucose-lowering drugs, and overtreatment as being at risk of overtreatment and being aged 80 years or above or living in a nursing home.

**Results:**

Among 460 participants, 36.0% received oral-antidiabetic drugs, 16.7% insulin, 16.2% both and 31.1% received diet/exercise. Overtreatment occurred in 12% of elderly patients with DM2, risk of overtreatment in 24%. Overtreatment was significantly associated with urban residency (OR 2.17). Female elderly patients with DM2 were significantly less often at risk of overtreatment (OR 0.59). Cluster effects were evident between general practices’ treatment and monitoring of elderly patients with DM2 in quantitative data.

**Conclusion:**

Overtreatment is a relevant problem in elderly patients with DM2 for which GPs should regularly check and start deprescribing. Cluster effects suggest heterogeneity between general practices in diabetes management and monitoring.

## KEY MESSAGES

In our study in German general practice, 12% of elderly patients with DM2 were overtreated, 24% were at risk of overtreatment.

GPs should regularly check for overtreatment and consequently start deprescribing.

Policymakers may support these efforts by addressing overtreatment in chronic care programs and by ensuring adequate reimbursement.

## Introduction

The management of diabetes type 2 (DM2) involves controlling hyperglycaemia to prevent microvascular and macrovascular complications [1,2]. Therefore, guidelines recommend life style changes, diabetes self-management education (DSME, Box 1) [1–3], disease management programs (DMP, Box 1) [1]_,_ and, if necessary, glucose-lowering drugs [1,3]. Observational studies have reported suboptimal DM2 control in routine care [4]. Guidelines advised against targeting a haemoglobin A1c (HbA1c) <47.5 mmol/mol (6.5%) in elderly patients with DM2, regardless of their frailty or comorbidities, as harms outweigh the benefits [1–3]. A low HbA1c results in increased hypoglycaemia, which is associated with increased falls, cardiovascular and cerebral events and a lower life expectancy in elderly patients with DM2 [3,5,6]. In those, several studies have described overtreatment in routine care [7–9]. A Norwegian study in general practice found potential overtreatment in 12% of elderly patients with DM2 [9]. A US study and the GUIDANCE study, reported overtreatment in 26% versus 44.7% elderly patients with DM2 [8,10]. A Spanish study showed that elderly patients with DM2 were more likely to achieve their HbA1c targets than younger patients, but were also more frequently treated with insulin monotherapy [11].

For general practitioners (GPs) applying quaternary prevention is crucial [9,12]. This involves identifying elderly patients with DM2 who are at risk of overtreatment or who are overtreated [12,13] and also to balance possible harms and risks. Therefore, our study aim was to assess the metabolic control, and diabetes treatment regimens and associated variables of elderly patients with DM2 in general practice. Consequently, our research question was: How is the metabolic control in elderly patients with DM2 and how are they treated in general practice in the German federal state Saxony-Anhalt?

## Methods

### Study design

This is a cross-sectional study of diabetes treatment in elderly patients with DM2 in general practice in Saxony-Anhalt, a federal state of Germany.

### Recruitment

For this study we contacted all GPs throughout Saxony-Anhalt by fax and 47 general practices agreed to participate. In three of these, GPs were subspecialists in diabetology. We excluded one general practices from further analysis due to missing data, leaving 46 general practices for our analyses.

### Sample selection

Participating general practices created a numbered list of all patients who met the following inclusion criteria: Diagnosis of DM2, age of 70 years or above, no palliative treatment and at least one practice contact within the last six months. From June 2019 to January 2020, our study nurse randomly selected 10 elderly patients with DM2 using Random Number Generator App and extracted their data from electronic health records (EHR). We entered pseudonymised patient data into a data mask without direct patient contact.

### Measurements

#### Treatment

We categorised the treatment regimen as either (1) with glucose-lowering drugs or (2) diet/exercise treatment. The group treated with glucose-lowering drugs included elderly patients with DM2 treated with oral antidiabetic drugs (OAD), any form of insulin therapy, and a combination of both. In the diet/exercise group, elderly patients with DM2 were treated with diet/exercise alone.

We assessed measures of long-term patient care and secondary prevention in both groups as followed: Current DMP enrolment, participation in DSME within the last 4 years, referrals for retinopathy screening and diabetes-related neuropathy screening within the last 12 months.

#### Assessment of HbA1c

We retrieved the most recent HbA1c monitoring within the last 24 months from EHR. We categorised HbA1c according to the proposal of the Diabetes Working Group of the German Society for Geriatrics [16]: <47.5 mmol/mol (6.5%); 47.5–53.01 mmol/mol (6.5–7.0%); 53 mmol/mol − 69.4 mmol/mol (7.01–8.5%); >69.4 mmol/mol (>8.5%) [16].

#### Overtreatment and undertreatment

We defined risk of overtreatment as having an HbA1c <47.5 mmol/mol (6.5%) and receiving glucose-lowering drugs and overtreatment as being at risk of overtreatment and being aged 80 years or above or living in a nursing home [2,14–16]. We defined undertreatment as having an HbA1c value ≥ 69.4 mmol/mol (8.5%) or inadequate uptake of retinopathy or neuropathy screening [14,16].

#### Practice level factors

We used GP’s sex, general practices’ location (rural or urban: communities with less or more than 20,000 inhabitants) and GP’s medical specialisation as contextual factors. Practicing GPs in Germany have different specialisations: some specialise in general practice (sGP), others in internal medicine (iGP), but all are GPs. ‘GP’ refers to both.

#### Sociodemographic and disease-related factors

We considered sociodemographic variables as covariates: sex, age and residence at home or in a nursing home and included disease-related characteristics as: receiving home visits by a GP or a nursing service care and referral to a diabetes specialist within the past 12 months.

#### Statistical analysis

We determined the intraclass correlation coefficient (ICC) to describe correlations of diagnosis, monitoring and therapy on elderly patients with DM2 within the same general practices. ICC values <10% indicate small cluster effects, 10% to 25% indicate medium, and >25% indicate strong effects [20]. We used bivariate, multilevel logistic models to investigate the dependence of diagnostic and treatment monitoring variables on sociodemographic, disease-related and GP-level factors and determined odds ratios (ORs) and 95%-confidence intervals (CI). A significant likelihood ratio test indicates systematic differences in the management of DM2 between general practices. The analyses were performed with Stata version 16.1 (Stata Corp., College Station, TX) and R Statistical Software (v4.4.1 Development Core Team, Vienna, Austria, 2024).

#### Ethics approval

Participating GPs gave their informed consent to participate in the study. Patient data were extracted in an anonymous form which does not require individual patient consent in the German health services research. The ethics committee of the Martin Luther University of Halle-Wittenberg approved this study in April 2019 (reference number: 2018-170 BADIA I) and stated the commission had no ethical concerns about the feasibility of the study.

## Results

### Characteristics of study cohort and treating GP

Out of 1065 general practices contacted, 46 agreed to participate in our study (practice response rate 4.3%). Of the 460 elderly patients with DM2 studied, median age was 80 years, 59.3% were women (Table 1). While 9.6% lived in a nursing home, 27.4% received home visits from a GP or nursing service. Of the participating GPs, 76.1% were sGPs, while 23.9% were iGPs (Table 2).

**Table 1.** Characteristics of the study population of 460 elderly patients with DM2.

Characteristics	*n*	%	Median (range)	ICC
Sociodemographic and disease-related characteristics				
Sex (*n* = 460)				
Female	273	59.3		
Male	187	40.7		
Age (years) (*n* = 460)			80 (70–100)	
Residential situation (*n* = 459)				
Nursing home	44	9.6		
At home (alone or with relatives)	415	90.4		
sGP/iGP home visits or nursing service care (*n* = 460)				
Yes	126	27.4		
HbA1c (*n* = 429)			6.5 (4.9–12.5)	7.9%
<47.5 mmol/mol (6.5%)	206	48.0		
Between 47.5 mmol/mol (6.5%) and 53 mmol/mol (7.0%)	90	21.0		
Between >53 mmol/mol (7.0%) and 69.4 mmol/mol (8.5%)	113	26.3		
>69.4 mmol/mol (8.5%)	20	4.7		
Referral to diabetes specialist within last 12 months (*n* = 460)				13.5%
Yes	30	6.5		
Diagnostics and therapy monitoring				
Currently enrolled within DMP (*n* = 460)				33.7%
Yes	349	75.9		
Participation in DSME in last 4 years (*n* = 398)				42.1%
Yes	95	23.9		
Referral to retinopathy screening within the last 12 months (*n* = 460)				17.7%
Yes	196	42.6		
Neuropathy screening within the last 12 months (*n* = 460)				54.1%
Yes	236	51.3		
Treatment and therapy of diabetes mellitus (*n* = 456)				
Only insulin	76	16.7		19.2%
Only OAD	164	36.0		4.9%
Both insulin and OAD	74	16.2		8.4%
Only lifestyle modification diet and exercise	142	31.1		21.0%
Overtreatment * identified				
Yes	51	12.0		
Risk for overtreatment *(*n* = 425)				
Yes	102	24.0		

DMP: Disease Management Programme; DSME: Structured Diabetes Self-Management Education; DM2: Type 2 diabetes mellitus; GP: General practitioner; sGP: GP specialised in general practice; iGP: Internist GP, GP specialised as internist but working as GP; HbA1c: Glycated haemoglobin c; n: Absolute frequencies; %: Relative frequencies; ICC: Intraclass correlation, ICC values <10% indicate small cluster effects, 10% to 25% indicate medium, and >25% indicate strong effects [20]; OAD : Oral antidiabetic drug. Cluster effects remained robust despite controlling for various sociodemographic, patient-, and practice-related variables.

**Table 2.** Characteristics of the 46 sGPs/iGPs and their practices included in the study.

Characteristics	*n*	%
Sex of the attending GP (*n* = 46)		
** **Female	21	45.7%
** **Male	25	54.4%
Practice location (*n* = 46)		
** **Rural area (≤ 20.000 inhabitants)	27	58.7%
** **Urban area (> 20.000 inhabitants)	19	41.3%
Medical specialisation in general practice (*n* = 46)		
** **sGP	35	76.1%
** **iGP	11	23.9%

n: Absolute frequencies; %: Relative frequencies; sGP: Specialised GP, GP specialised in general practice; iGP: Internist GP, GP specialised as internist but working as GP.

### Treatment of elderly patients with DM2 and associated variables

Elderly patients with DM2 were treated with diet/exercise (31.1%), 36.0% with OAD only, 16.7% with insulin and 16.7% with both insulin and OAD (Table 1). Insulin treatment was more frequently in those with a Hb1c ≥47.5 mmol/mol (6.5%), in those living in a nursing home and in those with a referral to a diabetologists (Table S1). Treatment with both insulin and OAD was more frequent among elderly patients with DM2 with an Hb1c ≥ 47.5 mmol/mol (6.5%, ORs between 5.19 and 9.69, Table S1). Diet/exercise treatment was less frequent among those with an HbA1c ≥ 47.5 mmol/mol (6.5%; ORs between 0.10 and 0.19, Table S1) compared to elderly patients with DM2 with an HbA1c <47.5 mmol/mol (6.5%; Table S1). Elderly patients with DM2 receiving home visits or nursing care participated significantly less frequently in DMP, DSME, retinopathy and neuropathy screening (Table S2). Those with HbA1c level between ≥ 47.5 to 53 mol/mol (6.5% to 7.0%; OR 2.19) participated more frequently in DMP and neuropathy screening (OR 0.59; Table S2).

We found significant cluster-effects of DM2 treatment and monitoring on practice level (Table 1). These cluster effects remained after adjustment for practice and patient characteristics (Table S1 and Table S2). Female GPs were more likely to prescribe diet/exercise treatment (OR 2.52; Table S1).

### Metabolic control and variables associated with metabolic control

**Figure 1.**
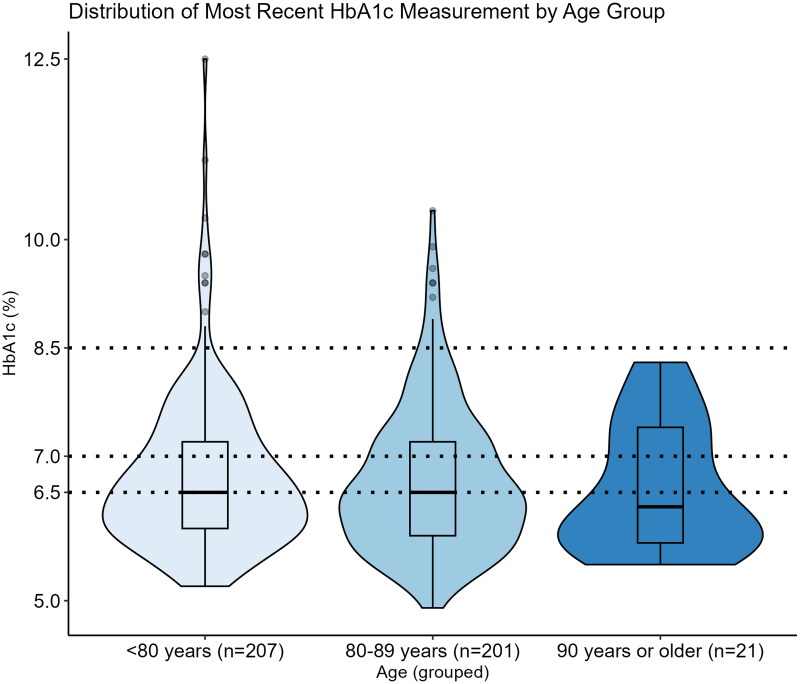
Distribution of most recent HbA1c measurement by age group. *Note*: Dotted lines represent the following cut-off values for HbA1c: 6.5% ≙ HbA1c of 47.5 mmol/mol; 7.0% ≙ HbA1c of 53 mmol/mol; 8.5% ≙ HbA1c of 69.4 mmol/mol.

**Figure 2.**
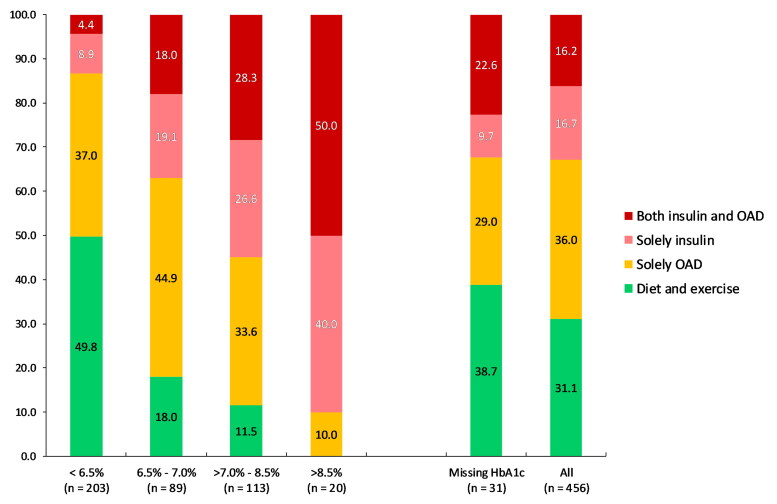
Distribution of diabetes medication by HbA1c (categorised). *Note*: HbA1c: Haemoglobine A1c; OAD: Oral diabetes medication; *n*: Number.

**Figure 3.**
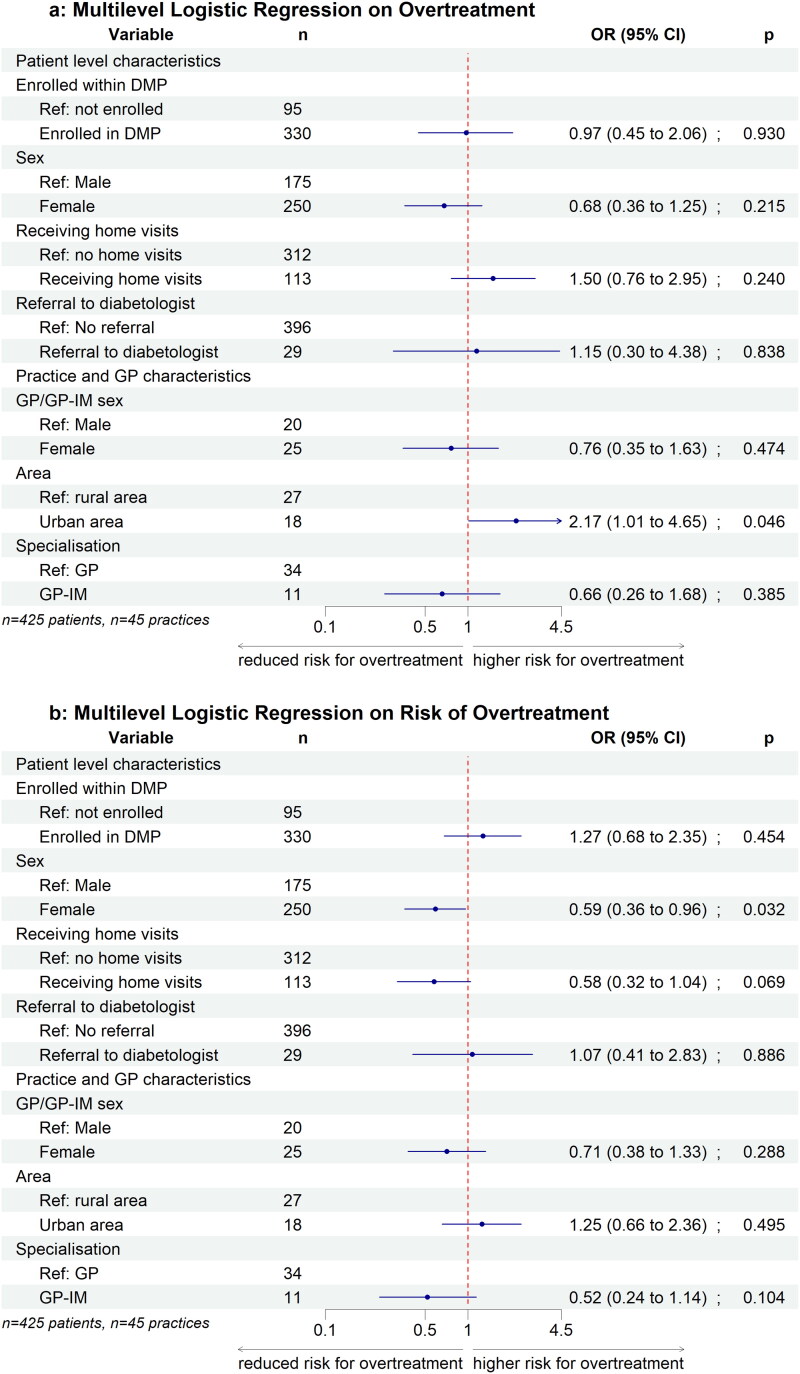
Multilevel logistic regression on overtreatment and risk of overtreatment. *Note*: *n*_patients_ = 425; *n*_practices_ = 45; (a) Overtreatment was defined by the simultaneous fulfilment of the following three criteria: HbA1c <47.5 mmol/mol (6.5%), treatment with oral antidiabetic drugs or insulin, and age ≥ 80 years or residence in a nursing home, (b) risk of overtreatment was defined as HbA1c <47.5 mmol/mol (6.5%), treatment with oral antidiabetic drugs or insulin.

In all age groups, 50% of elderly patients with DM2 had a HbA1c <47.5 mmol/mol (6.5%; Figure 1). Median of HbA1c was lowest in the group aged 90 years and older. In elderly patients with DM2 studied, 48.0% had an HbA1c <47.5 nmol/mol, but 50.2% of them were still on glucose-lowering drugs (Figure 2). Only 20 patients (4.7%) had an HbA1c ≥ 69.4 mol/mol (8.5%, Table 1). Risk of overtreatment was present in 24% of elderly patients with DM2, overtreatment in 12% of them (Table 1). Among overtreated elderly patients with DM2, 64.7% were treated with OAD, 25.5% with insulin and 9.8% with both (Table 3). Overtreatment was associated with urban residency in multilevel logistic regression (OR 2.17, Figure 3a). Female elderly patients with DM2 were less likely to be at risk of overtreatment (OR 0.59, Figure 3b).

**Table 3.** Characteristics of elderly patients with DM2 with overtreatment and without overtreatment.

	Overtreatment (*n* = 51)		No Overtreatment (*n* = 374)			
Characteristics	*n*	%	*n*	%	*p*	V
Sex					0.196	0.06
** **Female	25	14.3	150	85.7		
** **Male	26	10.4	224	89.6		
Age					N/A	
** **<80 years	1	0.5	204	99.5		
** **80–89 years	18	24.1	151	75.9		
** **90 years or older	2	9.5	19	90.5		
Residential situation					0.116	0.09
** **Nursing home	8	21.0	30	79.0		
** **At home (alone or with relatives)	43	11.1	344	88.9		
GP home visits/nursing care					0.280	0.06
** **No	34	10.9	278	89.1		
** **Yes	17	15.0	96	85.0		
Referral to diabetes specialist					0.972	0.01
** **Not within last 12 months	48	12.1	348	87.9		
** **Referral within last 12 months	3	10.3	26	89.7		
Currently enrolled within DMP					0.749	0.01
** **Yes	39	11.8	291	88.2		
** **No	12	12.6	83	87.4		
Participation in DSME in last 4 years	45		320		0.664	0.02
** **Yes	12	13.3	78	86.7		
** **No	33	12.0	242	88.0		
Referral to retinopathy screening	51		374		0.840	0.01
** **No referral within last 12 months	29	12.3	207	87.7		
** **Referral within last 12 months	22	11.6	167	88.4		
Neuropathy screening	51		374		0.724	0.01
** **No examination within last 12 months	25	12.4	176	87.6		
** **Examination within last 12 months	26	11.6	198	88.4		
Treatment	51		374		N/A	
** **Only insulin	13	17.8	60	82.2		
** **Only OAD	33	21.3	122	78.7		
** **Both Insulin and OAD	5	7.5	62	92.5		
** **No glucose-lowering drugs	–	–	130	100.0		

%: Frequency; DMP: Disease Management Programme; DSME: Structured Diabetes Self-Management Education; HbA1c: Glycated haemoglobin c; *n*: Absolute frequencies, *p*-values with respect to cluster-effects within general practices; V: Cramér’s V; %: relative frequencies. *Overtreatment was defined by the simultaneous fulfilment of the following three criteria: HbA1c < 47.54 mmol/mol (6.5%), treatment with oral antidiabetic drugs or insulin, and age ≥ 80 years or residence in a nursing home.

## Discussion

### Main findings

In this cross-sectional study 48.0% of elderly patients with DM2 studied had a HbA1c <47.5 mmol/mol, but 50.2% of them continued to receive glucose-lowering drugs. Using our definition of overtreatment and risk of overtreatment, 12% of elderly patients with DM2 were overtreated and additional 12% at risk of overtreatment. Overtreatment was significantly associated with urban residency and risk of overtreatment significantly negatively with female gender. Elderly patients with DM2 who received home visits or nursing care were less likely to participate in DMP, retinopathy or neuropathy screening or DSME, suggesting undertreatment. We found significant cluster effects indicating heterogeneity in management and monitoring of DM2 among general practices.

### Overtreatment

Overtreatment and risk of overtreatment in elderly patients with DM2 has been described in many European countries [7–9,17] in general practices. Our results stress that overtreatment remains a significant problem in elderly patients with DM2. Previous studied reported overtreatment in 9.8% to 62% of elderly patients with DM2 [5,7–9,17–19]. Our results tend towards the lower end of this wide range. Differences may be due to different study settings and population selection criteria [8,9,17–19], but heterogeneity also occurs in the definition of overtreatment [9]. We defined overtreatment and risk of overtreatment based on HbA1c targets established by the Diabetes Working Group of the German Society for Geriatrics and German guidelines, as these best reflected routine care in German general practice during 2019/2020 and focused on elderly patients with DM2, a patient group who is most affected by adverse effects of hypoglycaemia [14,16], At the time, the German guidelines recommended a HbA1c target between <47.54 and 57.38 mmol/l (<6.5–7.4%) [14]. While adjustments to the HbA1c target range were recommended for elderly patients with DM2 or those with reduced life expectancy, the recommendations for individualised target ranges remained vague [14]. Similarly, the NICE guideline recommended a HbA1c target range of 47.54–58.47 mmol/l (6.5–7.5%) for those with diet/exercise treatment or on glucose-lowering drug in 2019/2020 [2]. Physicians were encouraged to continue glucose-lowering drugs therapy even if HbA1c was <47.5 mmol/mol (<6.5%) in the absence of hypoglycaemia. Relaxed HbA1c targets were recommended for frail or elderly patients with DM2 [2]. American guidelines 2019/2020 argued for more liberal HbA1c targets in elderly patients with DM2, taking into account the importance of comorbidities, functional and cognitive status [15]. Elderly patients with DM2 should be classified (corresponding HbA1c targets) as healthy (<58.5 mmol/mol; 7.5%), complex/intermediate (<63.9 mmol/mol; <8.0%) and very complex (<69.4 mmol/l; <8.5%) [15].

We found female elderly patients with DM2 at higher risk of overtreatment. According to literature they also have a higher frequency of hypoglycaemias [7]. In contrast to our findings, other studies reported male elderly patients with DM2 to be more frequently at risk of overtreatment [20]. Because residency was rarely examined in studies on overtreatment in elderly patients with DM2, we were unable to compare our results with previous research. Residency and gender associations with overtreatment and risk of overtreatment, should be topics for further research.

Of elderly patients with DM2 studied, 48.0% had an HbA1c <47.5 mmol/mol (<6.5%), tighter than recommend in guidelines [2,14,15]. Consequences for those treated with diet/exercise would be to relax their diet/exercise regime. For those receiving glucose-lowering drugs, GPs should consider desprescribing, particularly for elderly patients with DM2 with a limited life expectancy, e.g. aged 80 years and above or those living in a nursing home [21,22]. This entails discussing treatment goals, benefits, risks, and patient preferences in a shared-decision making process [23]. However, elderly patients with DM2 may question the appropriateness to deprescribing and changing their medication habits, especially if they have not yet experienced side effects yet [24]. Physicians’ barriers may be lack of problem awareness or acceptance, reluctance to discontinue glucose-lowering drugs or adjust HbA1c targets, as well as organisational and time constrains [25]. To address these barriers, specific trainings for GPs may be beneficial. Checking for overtreatment should be mandatory in chronic care programs, including at least one medication review per year. DSME should include the harms of too-low HbA1c targets in elderly patients with DM2. To improve feasibility in busy general practices, future studies should assess if financial compensation for shared decision-making on HbA1c targeting could help prevent overtreatment.

### Undertreatment

As only 4.7% elderly patients with DM2 in our cohort had a HbA1c >69.4 mmol/mol, we perceive this number too small to draw any reasonable conclusion. In the overall cohort, secondary preventive procedures as DMP, DSME, retinopathy screening or foot examination decreased if elderly patients with DM2 received home visits or nursing care (Table S2). According to current guidelines, screening for diabetes complications in elderly patients with DM2 should be individualised and regularly reviewed [2,3,14]. However, this recommendation lacks specificity regarding when to stop screening for diabetes complications. Screening for diabetes complications is rarely studied in elderly patients with DM2 in real world general practices. Upon comparing our findings with studies among younger patients with DM2, we observed comparable frequencies for foot examinations [26,27] and retinopathy screenings [28].

### Variables associated with insulin treatment

The positive association of HbA1c and insulin treatment is described in previous studies and may be a part of therapy escalation [1]. Additionally, elderly patients with DM2 with nursing home residency may have a longer diabetes duration, therefore impaired insulin secretion [29]. However, studies showed that HbA1c is measured less frequently in nursing homes than recommended in guidelines [30]. Hence, our results could be due to inadequate monitoring and thus inadequate therapy adjustment.

The association between referral to diabetologists and insulin treatment is in concordance with previous studies [12,29] and may be explained by the higher disease burden of elderly patients with DM2 treated by diabetologist.

### Differences depending on GPs and practices

Our analyses revealed medium to strong cluster effects, suggesting, that therapy regime and monitoring are heterogeneous between general practices. Therefore, observed differences in DM2 treatment and monitoring cannot be adequately explained by patient population or variables studied. Cluster effects have previously been reported in younger patients with DM2 [31]. Therefore, studies in general practice should consider cluster effects between practices in their statistical analyses. As heterogeneity may also lead to additional cost, we believe that collaborative research projects with Statutory Health Insurances on how best to standardise DM2 treatment would be beneficial.

Our findings indicate that female GPs were more likely to treat with diet/exercise which is in concordance with international literature [32,33] and may be explained by gender differences in communication styles and beliefs about diabetes. Female GPs have been shown to engage more broadly in patient-centered interviewing including psychosocial issues [7,33].

### Strengths and limitations

This is a cross-sectional study on routine data on DM2 treatment in elderly patients in general practices. As we obtained data directly from EHR, bias due to patient-side memory or social desirability effects can be excluded. We included a large sample of 46 general practices, which allows for analyses of cluster effects. Few studies explored the association between practice-related and GP-related factors and DM2 therapy in elderly patients in general practice. We have attempted to control for these factors in our analyses.

One limitation is the low response rate of participating practices, which may be due time required for the practice staff to enable our study nurse the data extraction of an extensive variable set from EHR. Yet, it limits generalisation of our results. To more precisely assess overtreatment in future studies, patient reported outcomes such as hypoglycaemia, perceived health, medication side effects, additionally frailty, comorbidities and a more detailed categorisation of glucose-lowering drugs would be beneficial, but were unavailable in our database. Participants who attended DSME over 4 years ago were categorised as non-participants, limiting the conclusions that can be drawn from this variable. We were unable to address HbA1c progression and therefore limited our analyses to the most recent HbA1c available in the last 24 months. There may be cases where elderly patients with DM2 who had initially an HbA1c above target, received glucose-lowering drugs that lowered their HbA1c to <47.54 mmol/l (<6.5%) by the time of our data collection. However, this would still fall below guideline-recommended HbA1c targets, indicating overtreatment.

## Conclusion

Our findings indicate that overtreatment in elderly patients with DM2 in general practices remains a relevant problem, potentially causing harms and elevated costs. As DM2 management is a universal topic in Europe, our results might motivate studies on how to reduce overtreatment in routine general practices. GPs should regularly check for overtreatment and consequently start deprescribing. Policy may support these efforts by including checking for overtreatment in chronic care programs and adequate reimbursement. Substantial cluster effects were evident between general practices in treatment of elderly patients with DM2 treatment and monitoring. How best to reduce heterogeneity in DM2 treatment between general practices should be addressed in future studies.

## Supplementary Material

Supplemental Material
