# Supplementary material for: Potential overtreatment in elderly patients with diabetes mellitus: Results from a cross-sectional study in German general practice
Source: Eur J Gen Pract. 2025 Feb 18;31(1):2447723. doi: 10.1080/13814788.2024.2447723 (PMC11837937; doi:10.1080/13814788.2024.2447723)
Supplement: Supplemental Material [file IGEN_A_2447723_SM6179.docx]

# Supplemental

**Table S1: Multilevel logistic regression of the association between therapy and monitoring, sociodemographic and disease-related factors**

|  |  | Solely Insulin | | |  | Solely OAD | | |  | Both Insulin and OAD | | |  | Diet/excercise | | |
| --- | --- | --- | --- | --- | --- | --- | --- | --- | --- | --- | --- | --- | --- | --- | --- | --- |
|  |  | OR | 95%-C.I. | |  | OR | 95%-C.I. | |  | OR | 95%-C.I. | |  | OR | 95%-C.I. | |
| **Patient-level factors** |  |  |  |  |  |  |  |  |  |  |  |  |  |  |  |  |
| Enrolled within DMP (Ref: no) |  | 2.12 | [0.77; | 5.79] |  | 1.11 | [0.54; | 2.26] |  | 1.15 | [0.40; | 3.32] |  | 0.52 | [0.22; | 1.20] |
| Participation in DSME (Ref: no) |  | 0.73 | [0.30; | 1.80] |  | 1.13 | [0.64; | 2.00] |  | 1.01 | [0.45; | 2.30] |  | 0.71 | [0.33; | 1.54] |
| Retinopathy screening (Ref: no) |  | 1.60 | [0.78; | 3.27] |  | 1.40 | [0.84; | 2.32] |  | 0.74 | [0.37; | 1.50] |  | 0.65 | [0.35; | 1.23] |
| Neuropathy screening within 12 months (Ref: no) |  | 0.75 | [0.33; | 1.72] |  | 1.01 | [0.56; | 1.83] |  | 1.97 | [0.81; | 4.80] |  | 0.66 | [0.30; | 1.45] |
| Female (Ref.: male) |  | 0.88 | [0.45; | 1.71] |  | 0.71 | [0.44; | 1.15] |  | 0.97 | [0.49; | 1.93] |  | 1.68 | [0.94; | 3.02] |
| Age (z-Score) |  | 0.99 | [0.68; | 1.43] |  | 0.78 | [0.60; | 1.02] |  | 1.34 | [0.92; | 1.94] |  | 1.18 | [0.87; | 1.60] |
| Nursing home (Ref: at home) |  | **4.89** | **[1.57;** | **15.25]** |  | 0.45 | [0.13; | 1.55] |  | 0.40 | [0.09; | 1.69] |  | 0.60 | [0.18; | 1.98] |
| Home visits / nursing care (Ref: no) |  | 2.04 | [0.77; | 5.39] |  | 0.57 | [0.28; | 1.16] |  | 1.07 | [0.44; | 2.62] |  | 1.19 | [0.52; | 2.73] |
| HbA1c ≥ 47.5 mmol/mol (6.5%) to 53 mmol/mol (7.0 %) (Ref: < 47.5 mmol/mol, 6.5%) |  | **2.54** | **[1.02;** | **6.31]** |  | 1.39 | [0.77; | 2.51] |  | **5.19** | **[1.95;** | **13.82]** |  | **0.19** | **[0.09;** | **0.40]** |
| HbA1c > 53 mmol/mol (7.0 %) to 69.4 mmol/mol (8.5 %) (Ref: < 47.5 mmol/mol, 6.5%) |  | **5.06** | **[2.21;** | **11.60]** |  | 0.76 | [0.43; | 1.37] |  | **9.69** | **[3.92;** | **23.92]** |  | **0.10** | **[0.05;** | **0.24]** |
| HbA1c > 69.4 mmol/mol (8.5 %)  (Ref: < 47.5 mmol/mol, 6.5%) |  | **8.86** | **[2.15;** | **36.47]** |  | n.a. | (sparse data) | |  | n.a. | (sparse data) | |  | n.a. | (sparse data) | |
| Referral to diabetologist (Ref.: no) |  | **3.61** | **[1.11;** | **11.74]** |  | 0.41 | [0.14; | 1.22] |  | 1.79 | [0.54; | 5.97] |  | 0.61 | [0.17; | 2.18] |
| **Practice level factors** |  |  |  |  |  |  |  |  |  |  |  |  |  |  |  |  |
| GP/GP-IM sex female (Ref.: Male) |  | 0.97 | [0.39; | 2.43] |  | 0.72 | [0.43; | 1.20] |  | 0.57 | [0.28; | 1.17] |  | **2.52** | **[1.08;** | **5.90]** |
| Urban area (Ref.: rural area) |  | 1.12 | [0.45; | 2.78] |  | 0.89 | [0.53; | 1.48] |  | 0.67 | [0.32; | 1.40] |  | 1.37 | [0.60; | 3.13] |
| GP-IM (Ref.: GP) |  | 2.63 | [0.96; | 7.23] |  | 0.97 | [0.53; | 1.78] |  | 1.01 | [0.44; | 2.35] |  | 0.49 | [0.18; | 1.31] |
| **Variance components** |  |  |  |  |  |  |  |  |  |  |  |  |  |  |  |  |
| u_0j_^²^ (variance of intercept between practices) |  | 0.74 | [0.22; | 2.49] |  | 0.06 | [0.00; | 8.37] |  | n.a. | (sparse data) | |  | 0.81 | [0.27; | 2.39] |
| LR-test vs. single-level logistic model |  | χ²_(df = 1)_= 6.3; p < 0.01 | | |  | χ²_(df = 1)_= 0.19; p = 0.33 | | |  |  | | |  | χ²_(df = 1)_= 9.8; p < 0.01 | | |
| N Level-1 (patients) |  |  |  | 365 |  |  |  | 350 |  |  |  | 350 |  |  |  | 350 |
| N Level-2 (practices) |  |  |  | 42 |  |  |  | 42 |  |  |  | 42 |  |  |  | 42 |

Abbreviations ADM = antidiabetic medication; CI = confidence interval, DMP = Disease-Management-Programme; DSME = Structured Diabetes Self-Management Education; GP = general practitioner; sGP = specialised GP, GP specialised in general practice; iGP = internist GP, GP specialised as internist but working as GP; OR = Odds ratio, HbA1c = glycated haemoglobin c; n = absolute frequencies; % = relative frequencies; ICC = Intraclass correlation

**Table S2: Multilevel logistic regression of diabetes treatment and influencing factors**

|  |  | DMP | | |  | DSME | | |  | Retinopathy screening | | |  | Neuropathy screening | | |
| --- | --- | --- | --- | --- | --- | --- | --- | --- | --- | --- | --- | --- | --- | --- | --- | --- |
|  |  | OR | 95%-C.I. | |  | OR | 95%-C.I. | |  | OR | 95%-C.I. | |  | OR | 95%-C.I. | |
| **Patient-level factors** |  |  |  |  |  |  |  |  |  |  |  |  |  |  |  |  |
| Female (Ref.: Male) |  | 1.41 | [0.82; | 2.42] |  | 0.89 | [0.48; | 1.65] |  | 1.04 | [0.67; | 1.62] |  | 1.25 | [0.73; | 2.13] |
| Age (z-Score) |  | 0.89 | [0.66; | 1.22] |  | 0.83 | [0.59; | 1.17] |  | 0.95 | [0.74; | 1.21] |  | 0.95 | [0.71; | 1.28] |
| Nursing home (Ref: at home) |  | 1.39 | [0.45; | 4.28] |  | 0.82 | [0.15; | 4.50] |  | 1.15 | [0.43; | 3.08] |  | 0.98 | [0.31; | 3.11] |
| Home visits / nursing care (Ref: no) |  | **0.44** | **[0.21;** | **0.94]** |  | **0.19** | **[0.06;** | **0.59]** |  | **0.30** | **[0.15;** | **0.59]** |  | **0.38** | **[0.17;** | **0.85]** |
| HbA1c ≥ 47.5 mmol/mol (6.5%) to 53 mmol/mol (7.0 %) (Ref: < 47.54 mmol/mol, 6.5%) |  | **2.19** | **[1.02;** | **4.69]** |  | 1.06 | [0.45; | 2.48] |  | 1.07 | [0.60; | 1.92] |  | **2.59** | **[1.25;** | **5.36]** |
| HbA1c > 53 mmol/mol (7.0 %) to 69.4 mmol/mol (8.5 %) (Ref: < 47.5 mmol/mol, 6.5%)) |  | 1.35 | [0.69; | 2.63] |  | 1.31 | [0.61; | 2.80] |  | 1.48 | [0.85; | 2.55] |  | 1.71 | [0.88; | 3.34] |
| HbA1c > 69.4 mmol/mol (8.5 %)  (Ref: < 47.5 mmol/mol, 6.5%) |  | 4.39 | [0.78; | 24.71] |  | 1.82 | [0.40; | 8.23] |  | 0.68 | [0.22; | 2.11] |  | 1.18 | [0.33; | 4.16] |
| Referral to diabetologist (Ref.: no) |  | 1.47 | [0.48; | 4.52] |  | 1.56 | [0.48; | 5.05] |  | 1.15 | [0.48; | 2.75] |  | 1.57 | [0.54; | 4.53] |
| **Practice level factors** |  |  |  |  |  |  |  |  |  |  |  |  |  |  |  |  |
| GP/GP-IM female (Ref.: Male) |  | 1.75 | [0.64; | 4.77] |  | 0.50 | [0.15; | 1.61] |  | 1.84 | [0.93; | 3.63] |  | 1.15 | [0.29; | 4.49] |
| Urban area (Ref.: rural area) |  | 1.17 | [0.43; | 3.23] |  | 0.83 | [0.25; | 2.74] |  | 1.20 | [0.61; | 2.39] |  | 0.68 | [0.17; | 2.74] |
| GP-IM (Ref.: GP) |  | 0.74 | [0.23; | 2.38] |  | 1.20 | [0.32; | 4.56] |  | 0.57 | [0.26; | 1.28] |  | 3.05 | [0.61; | 15.27] |
| **Variance components** |  |  |  |  |  |  |  |  |  |  |  |  |  |  |  |  |
| u_0j_^²^ (variance of intercept between practices) |  | 1.78 | [0.79; | 4.05] |  | 2.36 | [1.02; | 5.44] |  | 0.72 | [0.32; | 1.63] |  | 4.30 | [2.20; | 8.41] |
| LR-test vs. single-level logistic model |  | χ²_(df = 1)_= 31.4;  p < 0.01 | | |  | χ²_(df = 1)_= 37.9;  p < 0.01 | | |  | χ²_(df = 1)_= 18.8;  p < 0.01 | | |  | χ²_(df = 1)_= 108.3; p < 0.01 | | |
| N Level-1 (patients) |  |  |  | 429 |  |  |  | 369 |  |  |  | 429 |  |  |  | 429 |
| N Level-2 (practices) |  |  |  | 45 |  |  |  | 42 |  |  |  | 45 |  |  |  | 45 |

Abbreviations ADM = antidiabetic medication; CI = confidence interval, DMP = Disease-Management-Programme; DSME = Structured Diabetes Self-Management Education; GP = general practitioner; sGP = specialised GP, GP specialised in general practice; iGP = internist GP, GP specialised as internist but working as GP; OR = Odds ratio, HbA1c = glycated haemoglobin c; n = absolute frequencies; % = relative frequencies; ICC = Intraclass correlation
